# Methods of EEG Signal Features Extraction Using Linear Analysis in Frequency and Time-Frequency Domains

**DOI:** 10.1155/2014/730218

**Published:** 2014-02-13

**Authors:** Amjed S. Al-Fahoum, Ausilah A. Al-Fraihat

**Affiliations:** ^1^Biomedical Systems and Informatics Engineering Department, Hijjawi Faculty for Engineering Technology, Yarmouk University, Irbid 21163, Jordan; ^2^Biomedical Engineering Department, Faculty of Engineering, Hashemite University, Zarqa 13115, Jordan

## Abstract

Technically, a feature represents a distinguishing property, a recognizable measurement, and a functional component obtained from a section of a pattern. Extracted features are meant to minimize the loss of important information embedded in the signal. In addition, they also simplify the amount of resources needed to describe a huge set of data accurately. This is necessary to minimize the complexity of implementation, to reduce the cost of information processing, and to cancel the potential need to compress the information. More recently, a variety of methods have been widely used to extract the features from EEG signals, among these methods are time frequency distributions (TFD), fast fourier transform (FFT), eigenvector methods (EM), wavelet transform (WT), and auto regressive method (ARM), and so on. In general, the analysis of EEG signal has been the subject of several studies, because of its ability to yield an objective mode of recording brain stimulation which is widely used in brain-computer interface researches with application in medical diagnosis and rehabilitation engineering. The purposes of this paper, therefore, shall be discussing some conventional methods of EEG feature extraction methods, comparing their performances for specific task, and finally, recommending the most suitable method for feature extraction based on performance.

## 1. Introduction

In recent years, brain computer interface and intelligent signal segmentation have attracted a great interest ranging from medicine to military objectives [1–6]. To facilitate brain-computer interface assembly, a professional method of feature extraction from EEG signal is desired.

**Figure 1:**
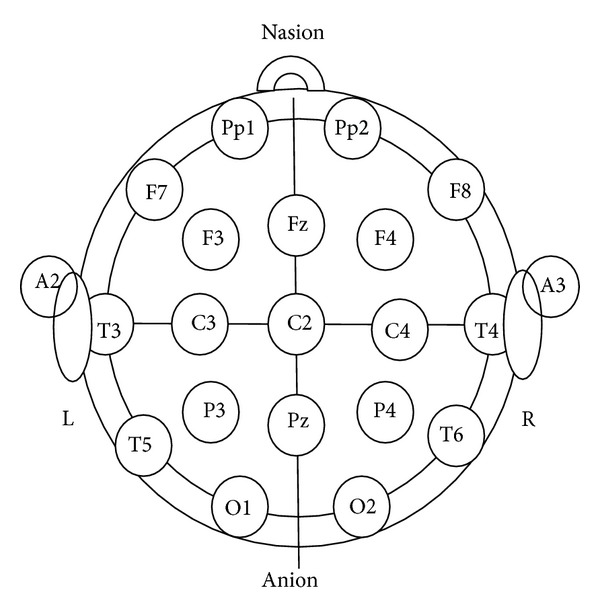
Standardized electrode placement scheme [11].

**Figure 2:**
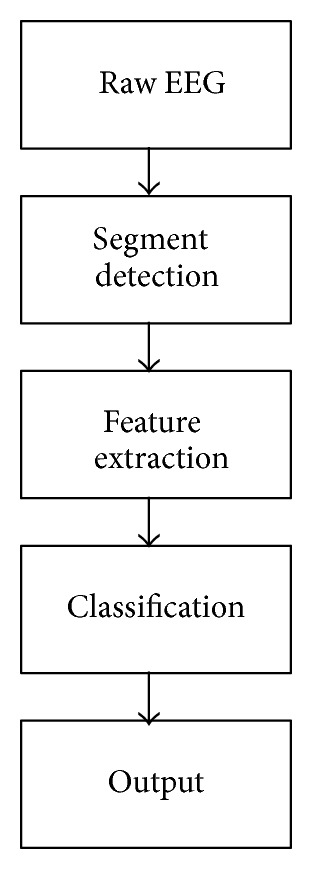
Stages of EEG signal processing.

The brain electrical activity is represented by the electroencephalogram (EEG) signals. Many neurological diseases (i.e., epilepsy) can be diagnosed by studying the EEG signals [7–9]. The recoding of the EEG signals is performed by fixing an electrode on the subject scalp using the standardized electrode placement scheme (Figure 1) [10–12]. However, there are many sources of artifacts. The signal noise which can set in when signal is being captured will adversely affect the useful feature in the original signal. The major sources of the artifact are muscular activities, blinking of eyes during signal acquisition procedure, and power line electrical noise [13]. Many methods have been introduced to eliminate these unwanted signals. Each of them has its advantages and disadvantages. Nevertheless, there is a common path for EEG signal processing (Figure 2). The first part is preprocessing which includes acquisition of signal, removal of artifacts, signal averaging, thresholding of the output, enhancement of the resulting signal, and finally, edge detection. The second step in the operation is the feature extraction scheme which is meant to determine a feature vector from a regular vector. A feature is a distinctive or characteristic measurement, transform, structural component extracted from a segment of a pattern [14]. Statistical characteristics and syntactic descriptions are the two major subdivisions of the conventional feature extraction modalities. Feature extraction scheme is meant to choose the features or information which is the most important for classification exercise [15–17]. The final stage is signal classification which can be solved by linear analysis, nonlinear analysis, adaptive algorithms, clustering and fuzzy techniques, and neural networks. This is done by exploiting the algorithmic characteristics of the feature vector of the data input and thus gives rise to a hypothesis [10, 15].

This paper presents a short review of mathematical methods for extracting features from EEG signals. The review considers five different methods for EEG signal extracting. The adopted approach is such that a full literature review is introduced for the five different techniques, summarizing their strengths and weaknesses.

## 2. Methods

Different articles were used to extract advantages and disadvantages of selected methods by thoroughly reviewing chosen articles including the main methods for linear analysis of one-dimensional signals in the frequency or time-frequency domain. Different common methods of interest were compared and the general advantages and disadvantages of these modalities were discussed.

### 2.1. Fast Fourier Transform (FFT) Method

This method employs mathematical means or tools to EEG data analysis. Characteristics of the acquired EEG signal to be analyzed are computed by power spectral density (PSD) estimation in order to selectively represent the EEG samples signal. However, four frequency bands contain the major characteristic waveforms of EEG spectrum [18].

The PSD is calculated by Fourier transforming the estimated autocorrelation sequence which is found by nonparametric methods. One of these methods is Welch's method. The data sequence is applied to data windowing, producing modified periodograms [19]. The information sequence *x*_*i*_(*n*) is expressed as

(1) xi(n)=x(n+iD), n=0,1,2,…,M−1while  i=0,1,2,…,L−1;

take *iD* to be the point of start of the *i*th sequence. Then *L* of length 2*M* represents data segments that are formed. The resulting output periodograms give

(2) $$\begin{array}{c} {\overset{\approx }{P}}_{xx}^{\left(i\right)}\left(f\right)=\frac{1}{MU}{\left|\sum_{n=0}^{M-1}x_{i}(n)w(n)e^{-j2\pi fn}\right|}^{2}. \end{array}$$

Here, in the window function, *U* gives normalization factor of the power and is chosen such that

(3) $$\begin{array}{c} U=\frac{1}{M}\sum_{n=0}^{M-1}w^{2}\left(n\right), \end{array}$$

where *w*(*n*) is the window function. The average of these modified periodograms gives Welch's power spectrum as follows:

(4) $$\begin{array}{c} P_{xx}^{W}=\frac{1}{L}\sum_{i=0}^{L-1}{\overset{\approx }{P}}_{xx}^{\left(i\right)}\left(f\right). \end{array}$$

### 2.2. Wavelet Transform (WT) Method

WT plays an important role in the recognition and diagnostic field: it compresses the time-varying biomedical signal, which comprises many data points, into a small few parameters that represents the signal [14].

As the EEG signal is nonstationary [7], the most suitable way for feature extraction from the raw data is the use of the time-frequency domain methods like wavelet transform (WT) which is a spectral estimation technique in which any general function can be expressed as an infinite series of wavelets [20–22]. Since WT allows the use of variable sized windows, it gives a more flexible way of time-frequency representation of a signal. In order to get a finer low-frequency resolution, WT long time windows are used; in contrast in order to get high-frequency information, short time windows are used [13].

Furthermore, WT only involves multiscale structure and not single scale. This method is just the continuation of the orthodox Fourier transform method [23]. Moreover, it is meant to resolve issues of nonstationary signals such as EEG [14]. In the WT method, the original EEG signal is represented by secured and simple building blocks known as wavelets. The mother wavelet gives rise to these wavelets as part of derived functions through translation and dilation, that is, (shifting) and (compression and stretching) operations along the time axis, respectively [24]. There are two categories for the WT; the first one is continuous while the other one is discrete [14].

#### 2.2.1. Continuous Wavelet Transform (CWT) Method

This can be expressed as

(5) $$\begin{array}{c} \text{CWT}\left(a,b\right)=\int_{-\infty }^{\infty }x\left(t\right)\psi_{a,b}^{*}\left(t\right)dt‍, \end{array}$$

*x*(*t*) stands for the unprocessed EEG, where *a* stands for dilation, and *b* represents translation factor. The *ψ*_*a*,*b*_(*t*) denotes the complex conjugate and can be calculated by

(6) $$\begin{array}{c} \psi_{a,b}\left(t\right)=\frac{1}{\sqrt{\left|a\right|}}\psi \left(\frac{t-b}{a}\right), \end{array}$$

where *ψ*(*t*) means wavelet. However, its major weakness is that scaling parameter *a* and translation parameter *b* of CWT change continuously. Thus, the coefficients of the wavelet for all available scales after calculation will consume a lot of effort and yield a lot of unused information [14].

#### 2.2.2. Discrete Wavelet Transform (DWT)

**Figure 3:**
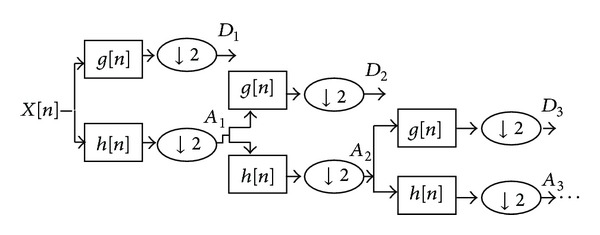
Implementation of decomposition of DWT [14].

In order to address the weakness of the CWT, discrete wavelet transform (DWT) has been defined on the base of multiscale feature representation. Every scale under consideration represents a unique thickness of the EEG signal [23]. The multiresolution decomposition of the raw EEG data *x*(*n*) is shown in Figure 3. Each step contains two digital filters, *g*(*n*) and *h*(*n*), and two downsamplers by 2. *The* discrete mother wavelet *g*(*n*) is a high pass in nature, while its mirror image is *h*(*n*) is a low-pass in nature.

As shown in Figure 3, each stage output provides a detail of the signal *D* and an approximation of the signal *A*, where the latest becomes an input for the next step. The number of levels to which the wavelet decomposes is chosen depending on the component of the EEG data with dominant frequency [14].

The relationship between WTs and filter *h*, that is, low pass, can be represented as follows:

(7) $$\begin{array}{c} H\left(z\right)H\left(z^{-1}\right)+H\left(-z\right)H\left({-z}^{-1}\right)=1. \end{array}$$

Here, *H*(*z*) represents filter's *h* *z*-transform. The high-pass filter's complementary *z*-transform is expressed as

(8) $$\begin{array}{c} G\left(z\right)=zH\left({-z}^{-1}\right). \end{array}$$

By precisely describing the features of the signal segment within a specified frequency domain and localized time domain properties, there are a lot of advantages that overshadow the high computational and memory requirement of the conventional convolution based implementation of the DWT [14, 23].

### 2.3. Eigenvectors

These methods are employed to calculate signals' frequency and power from artifact dominated measurements. The essence of these methods is the potential of the Eigen decomposition to correlate even artifact corrupted signal. There are a few available eigenvector methods, among them are Pisarenko's method, MUSIC method, and minimum-norm method [25, 26].

#### 2.3.1. Pisarenko's Method

Pisarenko's method is among the available eigenvector approaches used to evaluate power spectral density (PSD). To calculate the PSD, the mathematical expression *A*(*f*) is employed and given as [27, 28]

(9) $$\begin{array}{c} A\left(f\right)=\sum_{k=0}^{m}a_{k}e^{-j2\pi fk}‍. \end{array}$$

In the equation above, *a*_*k*_ stands for coefficients of the defined equation and *m* defines eigenfilter's *A*(*f*) order [25, 26]. Pisarenko method uses signal desired equation to estimate the signal's PSD from eigenvector equivalent to the minimum eigenvalue as follows:

(10) $$\begin{array}{c} P_{\text{PISARENKO}}=\frac{1}{{\left|A\left(f\right)\right|}^{2}}. \end{array}$$

#### 2.3.2. MUSIC Method

This method eradicates issues related to false zeros by the help of the spectra's average equivalent to artifact subspace of the whole eigenvectors [28]. Resulting power spectral density is therefore obtained as

(11) $$\begin{array}{c} P_{\text{MUSIC}}\left(f\right)=\frac{1}{\left(1/K\right){\sum_{i=0}^{K-1}\left|A\left(f\right)\right|}^{2}}. \end{array}$$

#### 2.3.3. Minimum Norm Method

This method makes false zeros in the unit circle to separate them from real zeros to be able to calculate a demanded noise subspace vector *a* from either the noise or signal subspace eigenvectors. However, while the Pisarenko technique form application of only the noise subspace eigenvector corresponding to the minimum eigenvalue, the minimum norm technique picks a linear combination of the whole of noise subspace eigenvectors [25, 26]. This technique is depicted by

(12) $$\begin{array}{c} P_{\text{MIN}}\left(f,K\right)=\frac{1}{{\left|A\left(f\right)\right|}^{2}}. \end{array}$$

All the aforementioned eigenvector methods can best address the signal that is composed of many distinctive sinusoids embedded in noise. Consequently, they are prone to yield false zeros and thus resulting in a relatively poor statistical accuracy [26].

### 2.4. Time-Frequency Distributions

These methods require noiseless signals to provide good performance. Therefore, very restricted preprocessing stage is necessary to get rid of all sorts of artifacts. Being time-frequency methods they deal with the stationary principle; windowing process is therefore required in the preprocessing module [29]. The definition of TFD for a signal *x*(*n*) was generalized by Cohen as [30]

(13) $$\begin{array}{c} P\left(t,w\right)=\frac{1}{2\pi }\int_{-\infty }^{\infty }\int_{-\infty }^{\infty }A\left(\theta ,\tau \right)\Phi \left(\theta ,\tau \right)e^{-j\theta t-j\omega \tau }d\theta \;d\tau ‍‍, \end{array}$$

where

(14) $$\begin{array}{c} A\left(\theta ,\tau \right)=\frac{1}{2\pi }\int_{-\infty }^{\infty }x\left(u+\frac{\tau }{2}\right)x^{*}\left(u-\frac{\tau }{2}\right)e^{j\theta u}du‍. \end{array}$$

*A*(*θ*, *τ*) is popularly known as ambiguity Function, and Φ(*θ*, *τ*) refers to kernel of the distribution, while *r* and *w* are time and frequency dummy variables, respectively.

Smooth pseudo-Wigner-Ville (SPWV) distribution is a variant method which incorporates smoothing by independent windows in time and frequency, namely, *W*_*w*_(*τ*) and *W*_*t*_(*t*) [29]:

(15) SPWV(t,w) =∫−∞∞Ww(τ)‍[∫−∞∞‍Wt(u−t)x(u+τ2)×x∗(u−τ2)du]e−jωτdτ.

The feature extraction using this method is based on the energy, frequency, and the length of the principal track. Each segment gives the values *E*_*k*_, *F*_*k*_, and *L*_*k*_. The EEG signal is firstly divided into *k* segments; then, the construction of a three-dimensional feature vector for each segment will take place. Energy of each segment *k* can be calculated as follows:

(16) $$\begin{array}{c} E_{k}=\int_{-\infty }^{\infty }\int_{-\infty }^{\infty }\vartheta_{k}\left(t,f\right)dt\;df‍‍, \end{array}$$

where *ϑ*_*k*_(*t*, *f*) stands for the time-frequency representation of the segment. However, to calculate the frequency of each segment *k*, we make use of the marginal frequency as follows:

(17) $$\begin{array}{c} F_{k}=\int_{-\infty }^{\infty }\vartheta_{k}\left(t,f\right)dt‍. \end{array}$$

Finally, for achieving good results, noiseless EEG signals or a well-denoised signal should be used for TFD [30].

### 2.5. Autoregressive Method

Autoregressive (AR) methods estimate the power spectrum density (PSD) of the EEG using a parametric approach. Therefore, AR methods do not have problem of spectral leakage and thus yield better frequency resolution unlike nonparametric approach. Estimation of PSD is achieved by calculating the coefficients, that is, the parameters of the linear system under consideration. Two methods used to estimate AR models are briefly described below [18, 19].

#### 2.5.1. Yule-Walker Method

In this method, AR parameters or coefficients are estimated by exploiting the resulting biased approximate of the autocorrelation data function. This is done by subsequently finding the minimization of the least squares of the forward prediction error as given below [31]:

(18) $$\begin{array}{c} \left[\begin{array}{cccc} {r\left(0\right)}_{xx} & {r\left(-1\right)}_{xx} & \cdots & {r(-p+1)}_{xx}\\ {r\left(1\right)}_{xx} & {r\left(0\right)}_{xx} & \ldots & {r(-p+2)}_{xx}\\ \vdots & \vdots & \ddots & \vdots \\ {r\left(p-1\right)}_{xx} & {r\left(p-2\right)}_{xx} & \cdots & {r\left(0\right)}_{xx} \end{array}\right]\times \left[\begin{array}{c} a\left(1\right)\\ a\left(1\right)\\ \vdots \\ a\left(p\right) \end{array}\right], \end{array}$$

where *r*_*xx*_ can be defined by

(19) $$\begin{array}{c} r_{xx}\left(m\right)=\frac{1}{N}\sum_{N=0}^{N-m-1}x^{*}\left(n\right)x\left(n+m\right)‍,\;m\geq 0. \end{array}$$

Calculating the above set of (*p* + 1) linear equations, the AR coefficients can be obtained:

(20) $$\begin{array}{c} P_{xx}^{BU}\left(f\right)=\frac{\sigma_{wp}^{2}}{{\left|1+\sum_{k=1}^{P}{\overset{⌢}{a}}_{p}(k)e^{-j2\pi fk}\right|}^{2}}, \end{array}$$

while ${\hat{\sigma }}_{wp}$ gives the approximated lowest mean square error of the *p*th-order predictor given as follows:

(21) $$\begin{array}{c} \sigma_{wp}^{2}=E_{p}^{f}=r_{xx}\left(0\right)\prod_{k=1}^{p}\left[1-{\left|a_{k}\left(k\right)\right|}^{2}\right]. \end{array}$$

#### 2.5.2. Burg's Method

It is an AR spectral estimation based on reducing the forward and backward prediction errors to satisfy Levinson-Durbin recursion [8]. Burg's method estimates the reflection coefficient directly without the need to calculate the autocorrelation function. This method has the following strength: Burg's method can estimate PSD's data records to look exactly like the original data value. It can yield intimately packed sinusoids in signals once it contains minimal level of noise.

The difference between method of Yule-Walker and Burg's method is in the way of calculating the PSD. For Burg's method, the PSD is estimated as follows:

(22) $$\begin{array}{c} P_{xx}^{BU}\left(f\right)=\frac{{\overset{⌢}{E}}_{p}}{{\left|1+\sum_{k=1}^{P}{\overset{⌢}{a}}_{p}(k)e^{-j2\pi fk}\right|}^{2}}. \end{array}$$

Parametric methods like autoregressive one reduce the spectral leakage issues and yield better frequency resolution. However, selecting the proper model order is a very serious problem. Once the order is too high, the output will induce false peaks in the spectra. If the order is too low, the result will produce smooth spectra [32].

## 3. Performance of Methods

The general aim of this review is to shed light on EEG signal feature extraction and to show how fast the method used for the signal extraction and how reliable it will be the extracted EEG signal features. Moreover, how these extracted features would express the states of the brain for different mental tasks, and to be able to yield an exact classification and translation of mental tasks. The speed and accuracy of the feature extraction stage of EEG signal processing are therefore very crucial, in order not to lose vital information at a reasonable time. So far in the discussed literature, wavelet method is introduced as a solution for unstable signals; it includes the representation by wavelets which are a group of functions derived from the mother wavelet by dilation and translation processes. The window with varying size is the most significant specification of this method since it ensures the suitable time frequency resolution in all frequency range [26]. Autoregression analysis suffers from speed and is not always applicable in real-time analysis while FFT appears to be the least efficient of the discussed methods because of its inability to examine nonstationary signals. The strength of AR method can be emphasized by further comparing its performance with that of classical FFT as shown in Table 1.

It is highly recommend to use AR method in conjunction with more conservative methods, such as periodograms, to help to choose the correct model order and to avoid getting fooled by spurious spectral features [32].

The most important application for eigenvectors is to evaluate frequencies and powers of signals from noise corrupted signal; the principle of this method is the decomposition of the correlation matrix of the noise corrupted. Three methods for eigenvectors module were discussed: Pisarenko, multiple signal classification (MUSIC), and minimum norm [27]. The good thing about the eigenvector method is that it produces frequency spectra of high resolution even when the signal-to-noise ratio (SNR) is low. However, this method may produce spurious zeros leading to poor statistical accuracy [26].

The TFD method offers the possibility to analyze relatively long continuous segments of EEG data even when the dynamics of the signal are rapidly changing. At the same time a good resolution both in time and frequency is necessary, making this method not preferable to use in many cases [30].

Table 2 shows the summary of advantages and disadvantages of the above-mentioned methods, their accuracies, speeds, and suitability to make it easier to compare their performances.

## 4. Conclusion

Five of the well-known methods for frequency domain and time-frequency domain methods were discussed. Acclaim about the definite priority of methods according to their capability is very hard. The findings indicate that each method has specific advantages and disadvantages which make it appropriate for special type of signals. Frequency domain methods may not provide high-quality performance for some EEG signals. In contrast, time-frequency methods, for instance, may not provide detailed information on EEG analysis as much as frequency domain methods. It is crucial to make clear the of the signal to be analyzed in the application of the method, whenever the performance of analyzing method is discussed. Considering this, the optimum method for any application might be different.

## Figures and Tables

**Table 1:** Comparison between FFT and AR [8].

Method	Frequency resolution	Spectral leakage
FFT	Low	High
AR	High	Low
WT	High	Low

**Table 2:** Comparison between performances of EEG methods.

Method name	Advantages	Disadvantages	Analysis method	Suitability
Fast fourier transform	(i) Good tool for stationary signal processing (ii) It is more appropriate for narrowband signal, such as sine wave (iii) It has an enhanced speed over virtually all other available methods in real-time applications	(i) Weakness in analyzing nonstationary signals such as EEG (ii) It does not have good spectral estimation and cannot be employed for analysis of short EEG signals (iii) FFT cannot reveal the localized spikes and complexes that are typical among epileptic seizures in EEG signals (iv) FFT suffers from large noise sensitivity, and it does not have shorter duration data record	Frequency domain	Narrowband, stationary signals

Wavelet transform	(i) It has a varying window size, being broad at low frequencies and narrow at high frequencies (ii) It is better suited for analysis of sudden and transient signal changes (iii) Better poised to analyze irregular data patterns, that is, impulses existing at different time instances	Needs selecting a proper mother wavelet	Both time and freq. domain, and linear	Transient and stationary signal

Eigenvector	Provides suitable resolution to evaluate the sinusoid from the data	Lowest eigenvalue may generate false zeros when Pisarenko's method is employed	Frequency domain	Signal buried with noise

Time frequency distribution	(i) It gives the feasibility of examining great continuous segments of EEG signal (ii) TFD only analyses clean signal for good results	(i) The time-frequency methods are oriented to deal with the concept of stationary; as a result, windowing process is needed in the preprocessing module (ii) It is quite slow (because of the gradient ascent computation) (iii) Extracted features can be dependent on each other	Both time and frequency domains	Stationary signal

Autoregressive	(i) AR limits the loss of spectral problems and yields improved frequency resolution (ii) Gives good frequency resolution (iii) Spectral analysis based on AR model is particularly advantageous when short data segments are analyzed, since the frequency resolution of an analytically derived AR spectrum is infinite and does not depend on the length of analyzed data	(i) The model order in AR spectral estimation is difficult to select (ii) AR method will give poor spectral estimation once the estimated model is not appropriate, and model's orders are incorrectly selected (iii) It is readily susceptible to heavy biases and even large variability	Frequency domain	Signal with sharp spectral features
